# Adiponectin Attenuates Lung Fibroblasts Activation and Pulmonary Fibrosis Induced by Paraquat

**DOI:** 10.1371/journal.pone.0125169

**Published:** 2015-05-06

**Authors:** Rong Yao, Yu Cao, Ya-rong He, Wayne Bond Lau, Zhi Zeng, Zong-an Liang

**Affiliations:** 1 Emergency Medicine Department, West China Hospital, Sichuan University, 37 Guoxue Road, Chengdu, Sichuan, 610041, PR China; 2 Emergency Medicine Department of Thomas Jefferson University Hospital, 1025 Walnut Street, 808 College Building, Philadelphia, PA, 19107, United States of America; 3 Respiratory Department, West China Hospital, Sichuan University, 37 Guoxue Road, Chengdu, Sichuan, 610041, PR China; University of Alabama at Birmingham, UNITED STATES

## Abstract

Pulmonary fibrosis is one of the most common complications of paraquat (PQ) poisoning, which demands for more effective therapies. Accumulating evidence suggests adiponectin (APN) may be a promising therapy against fibrotic diseases. In the current study, we determine whether the exogenous globular APN isoform protects against pulmonary fibrosis in PQ-treated mice and human lung fibroblasts, and dissect the responsible underlying mechanisms. BALB/C mice were divided into control group, PQ group, PQ + low-dose APN group, and PQ + high-dose APN group. Mice were sacrificed 3, 7, 14, and 21 days after PQ treatment. We compared pulmonary histopathological changes among different groups on the basis of fibrosis scores, TGF-β_1_, CTGF and α-SMA pulmonary content via Western blot and real-time quantitative fluorescence-PCR (RT-PCR). Blood levels of MMP-9 and TIMP-1 were determined by ELISA. Human lung fibroblasts WI-38 were divided into control group, PQ group, APN group, and APN receptor (AdipoR) 1 small-interfering RNA (siRNA) group. Fibroblasts were collected 24, 48, and 72 hours after PQ exposure for assay. Cell viability and apoptosis were determined via Kit-8 (CCK-8) and fluorescein Annexin V-FITC/PI double labeling. The protein and mRNA expression level of collagen type III, AdipoR1, and AdipoR2 were measured by Western blot and RT-PCR. APN treatment significantly decreased the lung fibrosis scores, protein and mRNA expression of pulmonary TGF-β_1_, CTGF and α-SMA content, and blood MMP-9 and TIMP-1 in a dose-dependent manner (p<0.05). Pretreatment with APN significantly attenuated the reduced cell viability and up-regulated collagen type III expression induced by PQ in lung fibroblasts, (p<0.05). APN pretreatment up-regulated AdipoR1, but not AdipoR2, expression in WI-38 fibroblasts. AdipoR1 siRNA abrogated APN-mediated protective effects in PQ-exposed fibroblasts. Taken together, our data suggests APN protects against PQ-induced pulmonary fibrosis in a dose-dependent manner, via suppression of lung fibroblast activation. Functional AdipoR1 are expressed by human WI-38 lung fibroblasts, suggesting potential future clinical applicability of APN against pulmonary fibrosis.

## Introduction

Paraquat (PQ) is a fast acting, effective herbicide used worldwide. Human exposure to PQ results in pulmonary accumulation of PQ, causing acute injury and ultimately fibrosis [1, 2]. Despite significant progress made in understanding the molecular mechanisms responsible for PQ-related lung fibrosis, the prognosis remains poor, due in part to lack of effective therapies [3, 4].

Adiponectin (APN), an adipocytokine predominantly secreted by adipocytes and found at high levels in plasma, has received great attention in the past decade for its anti-insulin resistance, anti-atherosclerotic and anti-inflammatory effects [5, 6, 7]. Several observations suggest to the potential role of APN against various fibrotic diseases. APN-knockout mice exhibit augmented carbon tetrachloride-induced liver fibrosis, while adenoviral-mediated increased APN expression prevented liver fibrosis in wild-type mice [8]. In a subtotal renal ablation model of APN-knockout mice, observed exacerbation of albuminuria and renal fibrosis was attenuated by adenoviral-mediated APN overexpression [9]. APN exerts protective effect against angiotensin II-induced cardiac fibrosis [10]. While evidence suggesting the anti-fibrotic effect of APN has accumulated, the effect of APN upon fibrotic processes in pulmonary tissue remains incompletely understood. Whether APN may attenuate PQ-induced lung fibrosis has never been previously investigated.

Therefore, the objectives of the current study are: 1) to determine whether APN exerts protective effect against PQ-induced pulmonary fibrosis; 2) to determine the effect of APN upon the expression of fibrosis-related cytokines and lung fibroblast activation; 3) to determine whether adiponectin receptors (AdipoR) are expressed by human lung fibroblasts; and if so, 4) if pulmonary fibroblast AdipoR are functional, and play a role in APN-mediated effects upon PQ-induced pulmonary fibrosis.

## Materials and Methods

This study was carried out in strict accordance with the ethical standards in the 1986 Directive 86/609/EEC, ''European Convention for the Protection of Vertebrate Animals Used for Experimental and other Scientific Purposes'' and the ''Guiding Principles in the Use of Animals in Toxicology'', adopted by the Society of Toxicology in 1989. This protocol was approved by the Committee on the Ethics of Animal Experiments at Sichuan University.

### Materials

PQ dichloride (1, 1′-dimethyl-4, 4′-bipyridinium dichloride) was purchased from Tokyo Kasei Kogyo Co., Ltd (Tokyo, Japan). Recombinant mouse globular APN isoform was from Adipobiotech (Beijing, China). Antibodies against transforming growth factor-β_1_ (TGF-β_1_), connective tissue growth factor (CTGF), α-smooth muscle actin (α-SMA), and collagen type III were all from Abcam (Boston MA, US). The enzyme linked immunosorbent assay (ELISA) kits of matrix metalloproteinase-9 (MIMP-9) and tissue inhibitor of metalloproteinase-1 (TIMP-1) were from USCN Life Science Inc (Wuhan, China) and Cusabio Life Science (Wuhan, China), respectively.

### Animals and treatment

Male BALB/c mice (Dossy Biological Technology Co. Ltd, Chengdu, China) were housed in a temperature (22±2°C) and humidity-controlled room with free access to fresh water and standard laboratory food. After 1 week of conditioning in a 12 hour light/dark cycle, 80 mice were randomly divided into four groups: 1) control group (saline injection), 2) PQ group (10 mg/kg delivered intraperitoneally, IP), 3) PQ + low-dose APN group (APN, 100 μg/kg by tail vein injection at 0, 24, and 48 hours after PQ exposure), 4) PQ + high-dose APN group (APN, 500 μg/kg by tail vein injection at 0, 24, and 48 hours after PQ exposure). Doses for high/low APN concentration were determined in preliminary experiments (data not shown). All reasonable efforts were made to ameliorate suffering, including anesthesia using pentobarbital (50 mg/kg, IP) for isolation of affected mice. Mice were monitored daily for signs of pain or distress. Moribund mice were humanely sacrificed as described below. Study design and biometric planning of each experiment was performed in accordance with a biostatistician. Mice were sacrificed for sample preparation by cervical dislocation after anesthesia. For each experiment, the single animal was an experimental unit. Serum/pulmonary samples were harvested at 3, 7, 14, and 21 days after PQ injection.

### Histopathological assessment of lung tissue

Lung tissue, fixed in 10% neutral-buffered formalin, was blocked in paraffin by an automated processor, and subjected to graded alcohol, xylene, and paraffin treatments. Sections of 4μm thickness were stained by hematoxylin and eosin (H&E) or Masson trichrome stain to assess lung injury/fibrosis extent. The severity of lung fibrosis was graded on a scale of 0 (normal lung) to 8 (most severe fibrotic lung), as previously reported by Ashcroft and colleagues [11]. At least 10 high-power fields (400 x) were evaluated per lung sample, scored independently by two investigators in blinded fashion.

### Cell culture and grouping

Human lung fibroblasts WI-38 obtained from Cell Bank of the Chinese Academy of Sciences (Shanghai, China) were cultured in DMEM supplemented with 10% fetal bovine serum (FBS; Gibco, Life Technologies), 100 UI/ml penicillin, and 100 μg/ml streptomycin (both from Cellgro, USA), and were incubated in a humidified incubator at 37°C in a 95% air/5% CO_2_ atmosphere until reaching confluence. From the 4th day onward, the culture medium was removed and replaced with fresh medium every 72 hours. Confluent cells were subsequently harvested for passage by a solution of trypsin (0.25%) and EDTA (0.02%) (Roche, Swiss).

When cells reached 80% confluence, they were randomly divided into four groups: Control group, PQ group, APN group, and AdipoR1 siRNA group. Lung fibroblasts WI-38 were incubated with 0.3 mmol/L PQ for 24 hours, with and without pretreatment with APN (5ug/mL) for 2 hours. The Adipo1 siRNA group cultured lung fibroblasts WI-38 were transfected with AdipoR1 siRNA via lipofectamine (Invitrogen) per manufacturer’s instructions, and then treated as per the APN group. Un-stimulated lung fibroblasts served as controls. Cells were collected for assays 24, 48, and 72 hours after PQ stimulation.

### Cell viability and apoptosis assay

Cell viability and apoptosis were measured by Kit-8 (CCK-8) (Beyotime, China) and fluorescein Annexin V-FITC/PI double labeling (Boehringer Mannheim GmbH), respectively, per manufacturer protocol.

For CCK-8 assay, growing cell density was adjusted to 5×10^4^ cells/ml. Cells were placed in 96-well dishes, incubated for 24 hours at 37°C in a 95% air/5% CO2 atmosphere, serum-starved for 24 hours in DMEM, transfected with reagents, and continuously incubated for 24 hours under routine conditions. After discarding supernatant, 10μl CCK-8 solution was added to each well. Cell culture continued for 1 hour. The OD of cells in the wells was quantitated at 450 nm via enzyme-linked immunometric meter (Thermo, USA).

For Annexin V-FITC/PI staining and flow cytometry, pulmonary fibroblasts were collected, washed with PBS, and adjusted to density 1×10^6^/ml. Cells were centrifuged at 500 rpm for 5 minutes at room temperature, and the supernatant was discarded. Cells were washed with PBS and stained with Annexin V/PI detection kit (Boehringer Mannheim GmbH) per manufacturer’s instructions. Both 5 μl Annexin V-FITC and 10 μl PI were added to 100 μl of suspended cells, and incubated for 15 minutes at room temperature in the dark. The samples were analyzed by FACS (Beckman Coulter Equipment) via Cell Quest Research Software (Becton Dickinson). To detect the same cell with two different lasers, the beams were separated from each other by a 40 ms time delay. A 15 mW argon ion laser emitting a 488 nm beam was the excitation source for Annexin V-FITC (FL1) and PI (FL2). Green fluorescence of Annexin V was collected with a 515 nm band pass filter; the red fluorescence of PI was collected with a 610 nm band pass filter.

### siRNA transfection

RNA interference was used to down-regulate the expression of AdipoR1 in lung fibroblasts. The siRNA were synthesized by Bioneer (Shanghai). The siRNA sequences used for targeted silencing of human AdipoR1 were 5′-ATCAATGTATCTTAAGGCGTGAATTCTCTAG-3′.

For gene knockdown experiments, lung fibroblasts WI-38 were plated in 6-cm dishes and cultured for 48 hours in DMEM containing 10% FBS. After incubating 24 hours in medium without FBS and antibiotics, cells were transfected with AdipoR1 siRNA via Lipofectamine 2000 (Invitrogen) per manufacturer’s instructions. After 48 additional hours of culture, cells were recultured in DMEM containing 10% FBS.

### Western blot

Western blot analysis was employed to detect the protein expression level of TGF-β_1_, CTGF, and α-SMA in lung tissue, and collagen type III, α-SMA, and AdipoR1/R2 in human lung WI-38 fibroblasts. Whole lung tissue homogenate or WI-38 lung fibroblasts lysates were prepared via lysis buffer [TianGen Biotech (Beijing) Co., Ltd.]. The lung tissue homogenate or cell lysates were separated by electrophoresis on 10% SDS-PAGE. Gels were transferred to a nitrocellulose membrane, blocked in 5% nonfat dry milk diluted in Tris-buffered saline. Blots were incubated overnight with anti-TGF-β_1,_ anti-CTGF, anti-α-SMA, anti-collagen type III, anti-AdipoR1, or anti-AdipoR2 antibodies (all from Abcam), at 4°C. Washed blots were incubated at room temperature for 60 minutes with a secondary biotinylated antibody (Zhongshan Golden Bridge Bio-technology, Beijing). After three 10 minute washings with TBST, membranes were analyzed by Western (Bio-Rad Laboratories Inc., Hercules, CA). Sample loadings were normalized by Western blot analysis with anti-GAPDH (Abcam).

### RT-PCR

We quantified mRNA expression in lung tissue by real-time polymerase chain reaction (RT-PCR). Total RNA was extracted from lung samples or lung fibroblasts via RNA Prep Pure [TianGen Biotech (Beijing) Co., Ltd.], and reverse transcribed into cDNA per manufacturer’s instructions. The quantity and quality of isolated RNAs were evaluated via absorbance measurements at 260 and 280 nm wavelengths. The mRNA expression level of TGF-β_1_, CTGF, and α-SMA in lung tissue, and α-SMA, collagen type III, and AdipoR1/R2 in human lung WI-38 fibroblasts were measured via RT-PCR by an ICycler IQ Real time PCR detection system (Bio-Rad Laboratories Inc., Hercules, CA). Primers designed are listed in Table 1.

**Table 1:** Primer sequences used for RT-PCR.

*Gene*	forward primer (5’–3’)	reverse primer (5’–3’)
**TGFβ**_**1**_	5'-AGGAGACGGAATACAGGGCT-3'	5'-CCACGTAGTAGACGATGGGC-3'
**CTGF**	5'-AGCGGTGAGTCCTTCCAAAG-3'	5'-TTCATGATCTCGCCATCGGG-3'
**α-SMA**	5′-GACAATGGCTCTGGGCTCTGTAA-3'	5′-ATGCCATGTTCTATCGGGTACTT-3'
**Col III**	5'-TGGTCCCCAAGGTGTCAAAG-3'	5'-GGGGGTCCTGGGTTACCATTA-3'
**AdipoR1**	5′-GCGATAACGAGGTGGAGTGT-3'	5′-GATGGGTCCAGATGTTGCCA-3'
**AdipoR2**	5′-ATAGGGCAGATAGGCTGGTTGA-3'	5′-GGATCCGGGCAGCATACA-3'

### Statistical analysis

All results are presented as mean±SEM. Comparisons between groups at each time point were made by one-way analysis of variance (ANOVA), followed by Student Newman—Keuls test. P values less than 0.05 were considered statistically significant.

## Results

### APN attenuates PQ-induced pulmonary fibrosis in dose-dependent manner

**Fig 1:**
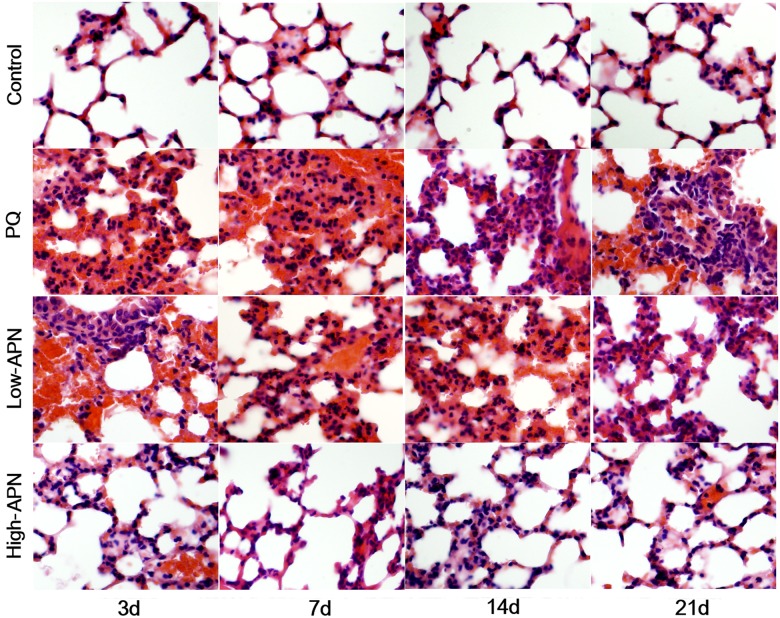
H&E stained histological evaluation of mouse lungs in the control, PQ, PQ + low-dose APN, and PQ + high-dose APN groups on days 3, 7, 14, 21 after PQ injection (×200 magnification). Compared with control group, PQ (10 mg/kg) intoxication showed progressively interstitial edema and inflammatory cell infiltration in the alveolar space and septum from day 3, reaching a peak on day 14. Both low high-dose (100 μg/kg) and high-dose (500 μg/kg) APN groups showed remarkably decreased interstitial edema and inflammatory cell infiltration, in a dose-dependent manner.

**Fig 2:**
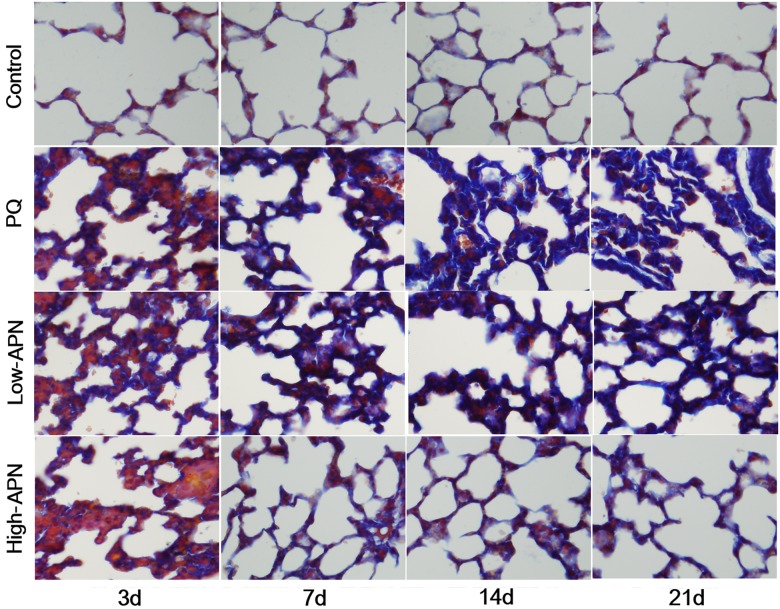
Histological evaluation of mice lungs in the control, PQ, PQ+ low-dose APN or PQ+ high-dose APN group on day 3, 7, 14, 21 after PQ injection with Masson stain (×200 magnification). Compared to control, PQ (10 mg/kg) exposure increased fibrosis (seen as blue collagen deposition) in the alveolar regions and small bronchioles from day 3, peaking on day 21. Both low-dose (100 μg/kg) and high-dose (500 μg/kg) APN groups exhibited decreased collagen deposition, in a dose-dependent manner.

Histologic tissue changes mediated by PQ and APN were evaluated by H&E or Masson trichrome staining. Compared to control (Fig 1), PQ exposure induced a marked inflammatory response characterized by interstitial edema and widespread inflammatory cell (lymphocytes and histiocytes) infiltration in the alveolar space and septum, substantially thickening and degrading normal alveolar structure by day 3. Masson trichrome stain revealed markedly increased collagen deposition, predominately in the thickened alveolar regions and small bronchioles (Fig 2), beginning day 3, and peaking day 21. APN treatment attenuated the marked interstitial thickening, inflammatory responses, and collagen accumulation induced by PQ in a dose-dependent manner.

Lung sections were randomized and scored blindly, assessed for interstitial thickening of alveolar or bronchiolar walls, collagen deposition, and damaged lung architecture. PQ exposure increased the lung fibrosis score assigned to histologic appearance (Table 2). APN treatment attenuated the lung fibrosis score in dose-dependent manner, particularly between days 14–21 (P<0.05).

**Table 2:** Lung fibrosis scores across groups.

	Day 3	Day 7	Day 14	Day 21
**control group**	**0**	**0**	**0**	**0**
**PQ group**	**3(2–5)**	**5(3–7)**	**7(5–8)** *	**8** *
**PQ + low-dose APN group**	**3(2–5)**	**4(2–5)**	**5(3–7)**	**6(4–7)** *
**PQ + high-dose APN group**	**2(1–4)**	**3(2–5)**	**3(2–5)** ^#^	**3(2–5)** ^#^

*: PQ group VS control group, *p<0*.*05*

^#^:PQ + APN group VS PQ group, *p<0*.*05*

### APN decreases PQ-induced TGF-β1, CTGF and α-SMA expression in lung tissue

**Fig 3:**
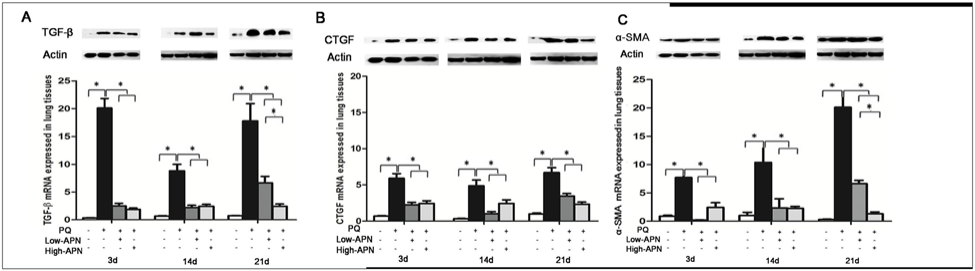
Effect of APN on TGF-β1, CTGF and α-SMA expression in lung tissues from control animals or PQ-exposed animals, treated with vehicle or APN. PQ (10 mg/kg, IP) increased, whereas APN (100 μg/kg or 500μg/kg, IV) revised, TGF-β1 (A), CTGF (B) and α-SMA (C) expression in mice lung tissues at 3, 14 and 21 d after PQ injection. The protein and mRNA expression were a determined via Western blot and RT-PCR, respectively. Values represent the mean ± standard error of the mean of four parallel measurements. *P<0.05. APN, adiponectin; TGF-β1, transforming growth factor-β1; CTGF, connective tissue growth factor; α-SMA, α-smooth muscle actin; PQ, paraquat.

To further investigate the specific anti-fibrotic effect of APN, the expression pattern of fibrosis-related cytokines in mouse lung tissue was evaluated by Western blot and RT-PCR. TGF-β_1_ has been demonstrated to be the most potent and direct stimulator of fibroblast proliferation and collagen production, and is requisite for pulmonary fibrosis of various etiologies [12]. We investigated whether the TGF-β_1_ signaling pathway is involved in the anti-fibrosis effect of APN upon PQ-induced lung fibrosis. As shown in Fig 3A, PQ exposure progressively increased TGF-β_1_ expression in lung tissue, while APN treatment decreased lung TGF-β_1_ protein and mRNA levels significantly at every measured time point (P<0.05) in dose-dependent fashion.

CTGF is considered both a downstream and cooperative mediator of TGF-β_1_, inducing fibroblasts to become myofibroblasts [13]. We analyzed the effect of APN upon pulmonary CTGF expression in mice subjected to PQ. PQ increased lung CTGF expression, which was significantly inhibited by both low and high-dose APN (P<0.05, Fig 3B).

TGF-β_1_ promotes myofibroblast differentiation and induces the expression of α-SMA in lung fibroblasts [12]. Increased expression of α-SMA is evidence of increased extracellular matrix (ECM)-producing myofibroblasts, an important feature of active fibrotic disease. We demonstrated PQ increased α-SMA expression in mouse lung tissue, and APN significantly inhibited PQ-mediated augmented α-SMA expression (P<0.05, Fig 3C). Together, these results indicate PQ stimulated myofibroblast transformation, a process blocked by APN.

### APN decreases PQ-induced elevation of MMP-9 and TIMP-1 in circulation

**Fig 4:**
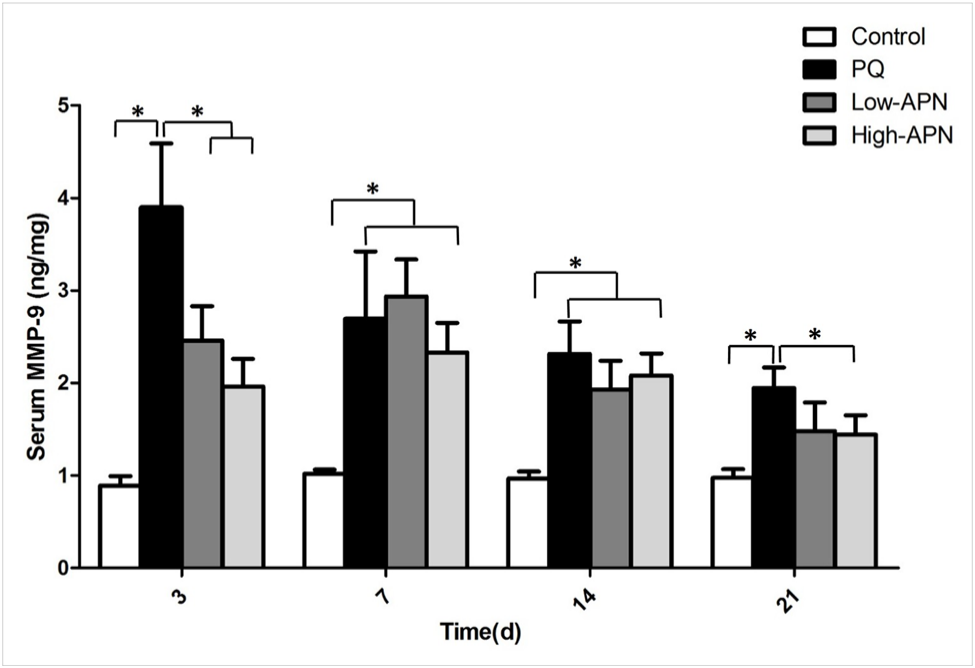
Effect of APN on MMP-9 blood levels in control animals or PQ-exposed animals, treated with vehicle or APN. PQ (10 mg/kg, IP) increased, whereas APN (100 μg/kg or 500μg/kg, IV) reversed, MMP-9 levels at 3, 7, 14 and 21 d after PQ injection. MMP-9 levels were determined by an enzyme-linked immunosorbent assay. Values represent the mean ± standard error of the mean of four parallel measurements. *P<0.05. APN, adiponectin; MMP-9, matrix metalloproteinase-9; PQ, paraquat.

**Fig 5:**
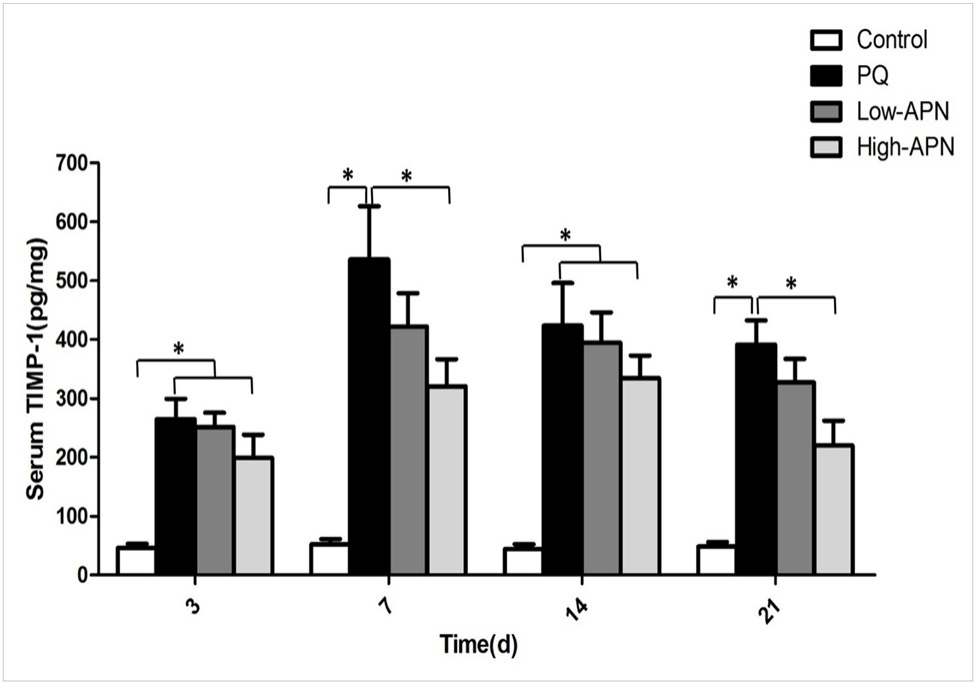
Effect of APN on TIMP-1 blood levels in control animals or PQ-exposed animals, treated with vehicle or APN. PQ (10 mg/kg, IP) increased, whereas APN (100 μg/kg or 500μg/kg, IV) reversed, TIMP-1 levels at 3, 7, 14and 21 d after PQ injection. TIMP-1 levels were determined by an enzyme-linked immunosorbent assay. Values represent the mean ± standard error of the mean of four parallel measurements. *P<0.05. APN, adiponectin; TIMP-1, tissue inhibitor of metalloproteinase-1; PQ, paraquat.

Of the matrix metalloproteinase members, MMP-9 is implicated in lung fibrosis [14]. TIMP-1, a physiological inhibitor of MMP-9, is upregulated during fibrosis [15, 16]. We determined the effect of APN upon expression/activity of both MMP-9 and TIMP-1. PQ exposure significantly increased MMP-9 and TIMP-1 blood levels, peaking on day 3 and 7 respectively. APN treatment significantly reduced this increase from day 3 after initial PQ exposure in a dose-dependent manner (Figs 4 and 5). It is noteworthy that both MMP-9 and TIMP-1 levels peaked on day 7 after APN treatment, suggesting a correction of imbalanced MMP-9/TIMP-1 ratio induced by PQ.

### APN attenuates PQ-induced cytotoxicity in human lung fibroblasts

**Fig 6:**
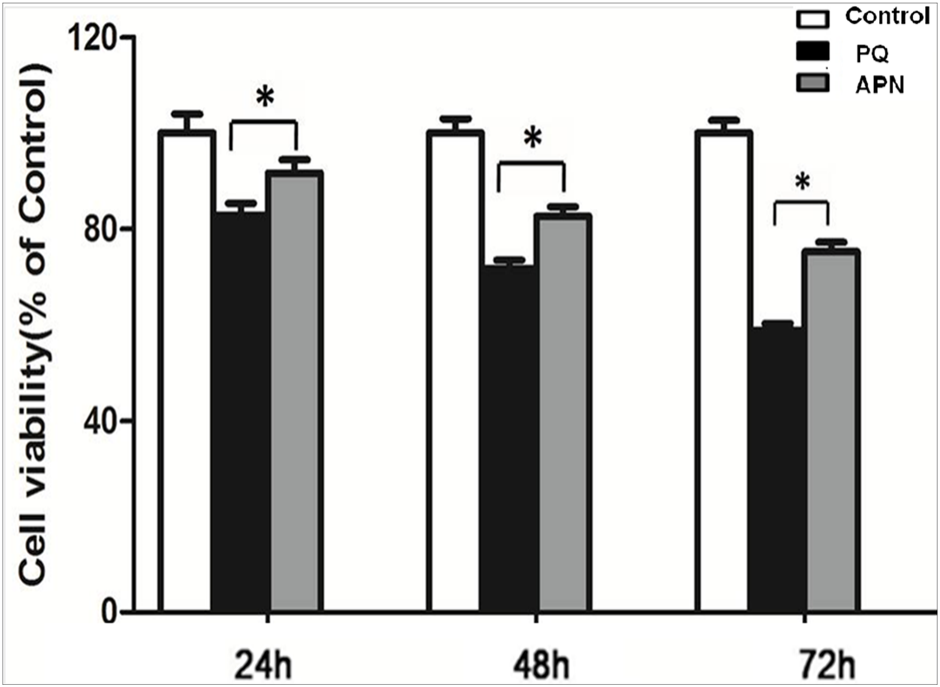
Effect of APN on cell viability in control or PQ-incubated lung fibroblast. PQ (0.3 mmol/L, 24 hours) decreased, whereas pretreated with APN (5 ug/mL, 2 hours) rescued, lung fibroblast viability at 24, 48 and 72 h after PQ incubation. Cell viability was measured via CCK-8 assay. Values represent the mean ± standard error of the mean of three parallel measurements. *P<0.05. APN, adiponectin; PQ, paraquat.

Fibroblasts are integral in lung fibrosis development [17]. We employed the normal human lung fibroblast cell line WI-38, to determine the effect of APN upon PQ-induced cytotoxicity. First, we measured cellular viability via CCK-8. Incubation with PQ (0.3 mmol/L, 24 hours) decreased lung fibroblast viability in time dependent manner. Pretreatment with APN (5 ug/mL, 2 hours) significantly increased cellular survival rate (P<0.05, Fig 6).

**Fig 7:**
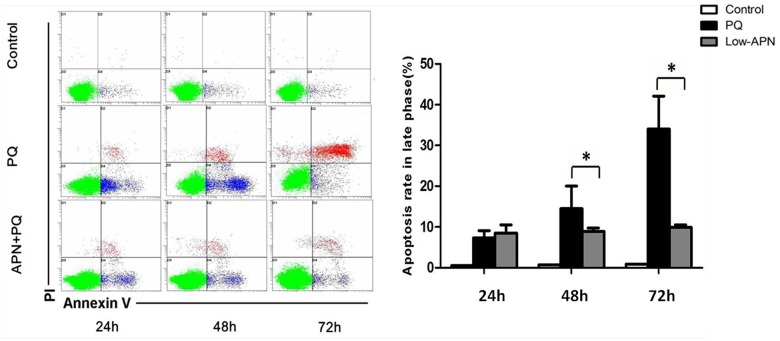
Effect of APN on cell apoptosis in control or PQ-incubated lung fibroblast. PQ (0.3 mmol/L, 24 hours) augmented, whereas pretreated with APN (5 ug/mL, 2 hours) rescued, lung fibroblast apoptotic rate at 24, 48 and 72 h after PQ incubation. Cell apoptosis was assessed by Annexin V-FITC/PI staining and flow cytometry. Values represent the mean ± standard error of the mean of three parallel measurements. *P<0.05. APN, adiponectin; PQ, paraquat.

Cellular apoptosis was assessed by Annexin V-FITC/PI staining and flow cytometry. Annexin V and PI negative cells (lower left quadrant) were considered nonapoptotic, viable cells. Dead cells are PI positive. Annexin V positive, but PI negative, cells are apoptotic. APN pretreatment (5 ug/mL, 2 hours) significantly decreased the apoptotic and dead population of lung fibroblasts subjected to PQ exposure (0.3 mmol/L, 24 hours) (Fig 7).

### APN decreases PQ-induced increased collagen type III expression in human lung fibroblasts

**Fig 8:**
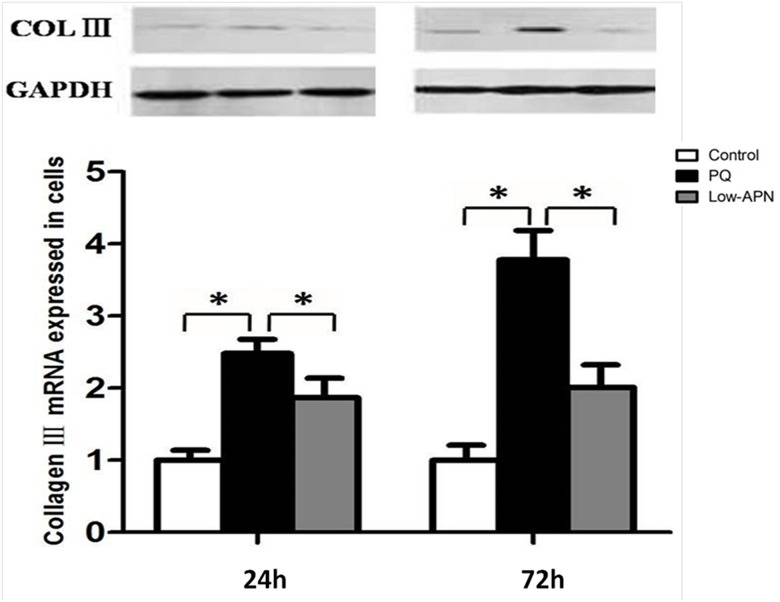
Effect of APN on collagen type III expression in control or PQ-incubated lung fibroblast. PQ (0.3 mmol/L, 24 hours) increased, whereas pretreated with APN (5 ug/mL, 2 hours) rescued, collagen type III expression in lung fibroblasts at 24 and 72 h after PQ incubation. The collagen type III protein and mRNA expression were a determined via Western blot and RT-PCR, respectively. Values represent the mean ± standard error of the mean of three parallel measurements. * P<0.05. APN, adiponectin; PQ, paraquat.

We specifically determined the effect of PQ on collagen type III expression, the main extracellular matrix component in pulmonary cells. Collagen type III deposition increases during acute lung injury and pulmonary fibrosis [18]. Employing Western blot and RT-PCR analysis, we demonstrated APN pretreatment (5 ug/mL for 2 hours) markedly inhibited PQ—induced collagen type III expression in human lung fibroblasts (P<0.05, Fig 8), suggesting PQ-induced ECM formation was significantly reversed by APN.

### APN receptor expression in lung fibroblasts WI-38

**Fig 9:**
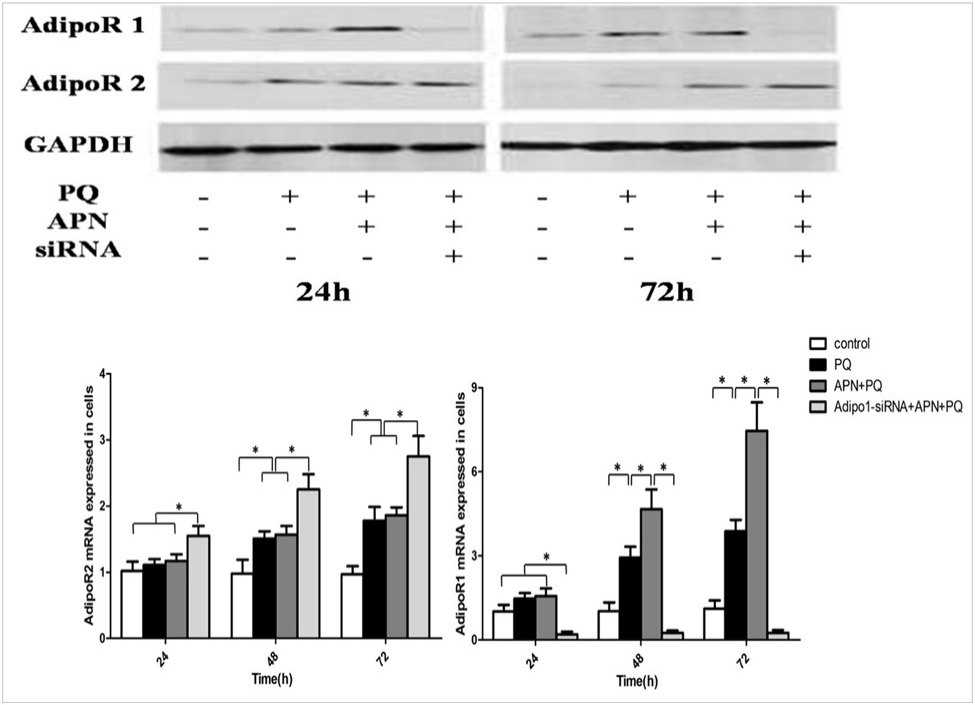
APN receptor expression in lung fibroblast. Both AdipoR1 and AdipoR2 expression were detected in WI-38 lung fibroblasts. PQ (0.3 mmol/L, 24 hours) treatments up-regulated protein and mRNA expression of both APN receptors in lung fibroblasts WI-38 at 24, 48 and 72 h after PQ incubation. APN (5 ug/mL, 2 hours) pretreatment further enhanced AdipoR1, but not AdipoR2, up-regulation. AdipoR1-specific siRNA successfully abrogated AdipoR1 expression. AdipoR1 and AdipoR2 expression were determined by Western blot and RT-PCR. Values represent the mean ± standard error of the mean of four parallel measurements. *P<0.05. APN, adiponectin; PQ, paraquat, AdipoR, APN receptor.

Previous studies indicate APN exerts anti-fibrotic effect by reducing inflammatory reaction or oxidative stress [8, 19]. We hypothesize APN influences PQ-mediated lung fibrosis directly via lung fibroblasts, a predominant effector cell in organ fibrosis. To test this, we determined whether purified populations of human lung fibroblasts WI-38 express the APN receptors (AdipoR1 and AdipoR2). Western blot and RT-PCR analysis demonstrate both AdipoR1 and AdipoR2 protein and mRNA are expressed in WI-38 lung fibroblasts. When cells were incubated with PQ for 24 hours, protein/mRNA expression of both AdipoR1 and AdipoR2 were up-regulated. APN pretreatment further enhanced AdipoR1, but not AdipoR2, up-regulation (Fig 9).

### AdipoR1-specific siRNA reverses the protective effect of APN against PQ-mediated fibrosis in lung fibroblasts WI-38

**Fig 10:**
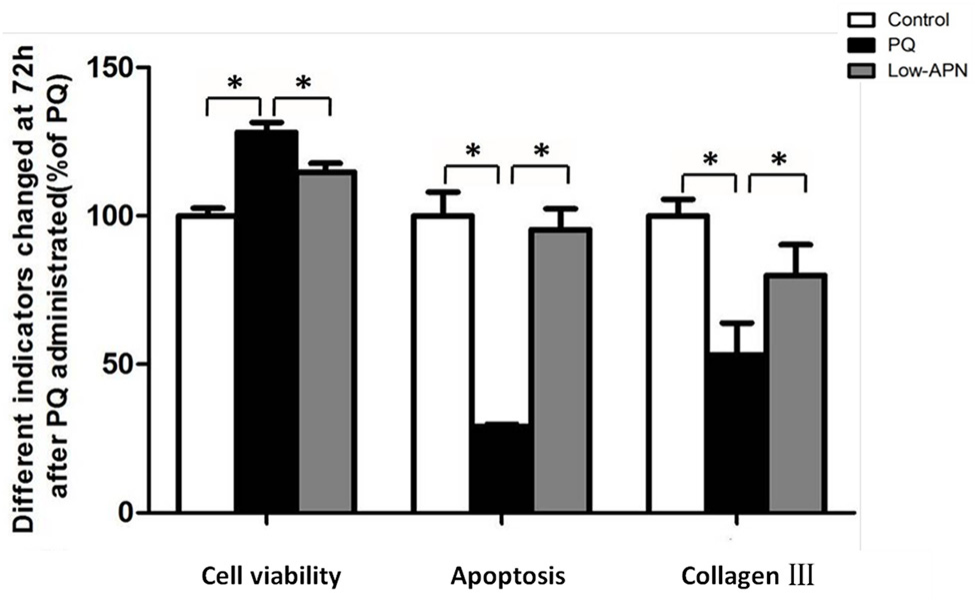
AdipoR1-specific siRNA reversed the protective effect of APN against PQ-mediated fibrosis in lung fibroblasts WI-38 at 72 h after PQ incubation. Cell viability, apoptosis and collagen type III mRNA expression in lung fibroblasts were measured via CCK-8 assay, Annexin V-FITC/PI staining with flow cytometry, and RT-PCR, respectively. Values represent the mean ± standard error of the mean of three parallel measurements. *P<0.05. APN, adiponectin; PQ, paraquat, AdipoR, APN receptor.

Experiment 3.6 suggests AdipoR1, not AdipoR2, may be involved in APN-mediated protective effect against PQ-mediated fibrotic effects. To determine the functionality of AdipoR1 expressed in lung fibroblasts, lung fibroblasts were transfected with AdipoR1-specific siRNA for 48 hours, and incubated with 5ug/mL APN for an additional 2 hours, and finally 0.3 mmol/L PQ for 24 hours. AdipoR1-specific siRNA successfully abrogated AdipoR1 expression (Fig 9). Knockdown of AdipoR1 significantly reversed the APN-mediated protective effect against PQ-mediated fibrotic effect (Fig 10). These data support the involvement of AdipoR1 expressed by human lung fibroblasts in APN-mediated protective effect against PQ-induced pulmonary fibrosis.

## Discussion

Pulmonary fibrosis is one of the most common and serious clinical sequela of PQ poisoning, responsible for high mortality [3, 4]. To date, no safe and effective treatments exist halting and/or reversing fibrotic pathophysiology. In the present study, we demonstrated APN, an adipocytokine with a wide range of biologic effects, exerts protective effect against PQ-induced pro-fibrogenic processes.

To discern the responsible underlying mechanisms for the observed phenomenon, we determined the expression pattern of fibrosis-related cytokines in mouse lung tissue. Of the relevant cytokines, TGF-β is the most important pro-fibrotic cytokine mediating myofibroblast formation and ECM production [11, 12, 20]. As excessive fibroproliferation and ECM accumulation are critical determinants of fibrogenesis, TGF-β inhibition has successfully reduced fibrosis development in different experimental models [21, 22]. CTGF has been implicated as both a downstream and cooperative mediator of TGF-β, inducing fibroblasts to become myofibroblasts [13]. CTGF also directly or indirectly enhances the expression of its own inducer, TGF-β, in positive feedback manner [23–25]. In our study, we demonstrated PQ increased expression of TGF-β_1_ and CTGF, which was significantly reversed by APN treatment, suggesting the anti-fibrotic effect of APN is mediated partly via inhibition of TGF-β_1_/CTGF signaling.

It is well established that the myofibroblast is the ultimate effector cell in pulmonary fibrosis. As a differentiated fibroblast, myofibroblasts are the key source of ECM and various inflammatory cytokines, including TGF-β_1_ [26], and are commonly identified by expression of α-SMA. In the current study, APN treatment inhibited fibroblast differentiation into myofibroblasts.

The expression of MMPs and their physiologic inhibitors TIMPs are integral to fibrotic tissue development [27, 28]. While many MMPs are purportedly involved in pulmonary fibrosis, MMP-9 is one of the most relevant pro-fibrotic members of the family [14]. Increased MMP-9 is observed in clinical and experimental models of fibrosis [29]. TIMP-1, an inhibitor of MMP-9, is up-regulated during fibrosis [15,16]. In the current study, marked increase of MMP-9 and TIMP-1 were observed in mice subjected to PQ exposure. Moreover, increased MMP-9 expression preceded increased TIMP-1 expression, the former peaking on day 3, and the latter peaking on day 7, a pattern that supports the notion an imbalance between matrix degradation and its inhibition may contribute to PQ-induced pulmonary fibrosis. APN treatment results in decreased MMP-9 and TIMP-1 levels, and simultaneous peaking of both proteins on day 7, indicating correction of PQ-induced imbalanced MMP-9/TIMP-1 ratio.

In human lung fibroblasts WI-38 subjected to PQ in vitro, PQ decreased cellular viability, and increased apoptosis. APN significantly decreased PQ-induced cytotoxicity. PQ also markedly increased collagen type III synthesis (a major ECM component), an effect inhibited by APN in dose-dependent fashion.

To test our hypothesis APN influences pulmonary fibrosis directly via lung fibroblasts, we confirmed the presence of AdipoR1 and AdipoR2 (the two known transmembrane APN receptors) in human lung fibroblasts WI-38 via Western and RT-PCR. APN up-regulated AdipoR1, but not AdipoR2, expression in lung fibroblasts exposed to PQ. Additionally, siRNA-mediated knockdown of AdipoR1 abrogated APN's protective effects against PQ-mediated fibrosis. Together, these data support the functionality and possible therapeutic potential of AdipoR1 against fibrosis development post-PQ exposure.

The current study is important for several reasons. Firstly, our data provides the first evidence that exogenously administered globular isoform of APN mitigates the PQ-mediated pulmonary fibrogenic response. Secondly, our data supports the responsible mechanism underlying APN-mediated anti-fibrotic effect may involve 1) inhibition of TGF-β_1_/CTGF signaling and 2) correction of the PQ-induced imbalance of MMP-9/TIMP-1 ratio, ultimately blocking the differentiation of fibroblasts into myofibroblasts. Thirdly, we demonstrate lung fibroblasts express functional AdipoR1, supporting the potential utilization of APN as an agent mitigating lung fibrosis. Our current works have some limitations. Considering the wide range of biologic effects of APN, there may be far more complex mechanisms than we had mentioned in this manuscript. For example, whether APN is involved in prevention of epithelial cells death, survival, transformation, or other important fibrosis related events, in context of PQ-induced fibrosis remain to be confirmed by further studies. In addition, mechanisms in PQ-induced lung injury could be different with that in other types of lung fibrosis, such as idiopathic pulmonary fibrosis.

In summary, we have demonstrated the pathogenicity of PQ upon pulmonary tissue in an animal model. In dose-dependent manner, APN mitigates PQ-mediated pulmonary fibrosis, via suppression of fibroblasts activation and differentiation. We present for the first time a novel therapeutic agent with exciting application for a clinical disease currently lacking efficacious therapy. Exploring the therapeutic and chemoprotective potentials of APN for treatment and prevention of pathologic pulmonary fibrosis in future toxicologic studies is warranted.

## References

[1] Dinis-Oliveira RJ, Duarte JA, Sanchez-Navarro A, Remiao F, Bastos ML, Carvalho F. Paraquat poisonings: mechanisms of lung toxicity, clinical features, and treatment. Crit Rev Toxicol. 2008; 38: 13–71. 18161502 10.1080/10408440701669959

[2] Hoet PH, Lewis CP, Demedts M, Nemery B. Putrescine and paraquat uptake in human lung slices and isolated type II pneumocytes. Biochem Pharmacol. 1994; 48: 517–524. 8068038 10.1016/0006-2952(94)90281-x

[3] Yamashita M, Yamashita M, Ando Y. A long-term follow-up of lung function in survivors of paraquat poisoning. Hum Exp Toxicol. 2000; 19: 99–103. 10773838 10.1191/096032700678815729

[4] Suntres ZE. Role of antioxidants in paraquat toxicity. Toxicology. 2002; 180: 65–77. 12324200 10.1016/s0300-483x(02)00382-7

[5] Berg AH, Combs TP, Scherer PE. ACRP30/adiponectin: an adipokine regulating glucose and lipid metabolism. Trends In Endocrinology And Metabolism. 2002; 13: 84–89. 11854024 10.1016/s1043-2760(01)00524-0

[6] Okamoto Y, Kihara S, Funahashi T, Matsuzawa Y, Libby P. Adiponectin: a key adipocytokine in metabolic syndrome. Clin Sci (Lond). 2006; 110: 267–278. 16464169 10.1042/CS20050182

[7] Robinson K, Prins J, Venkatesh B. Clinical review: adiponectin biology and its role in inflammation and critical illness. Crit Care. 2011; 15: 221 10.1186/cc10021 21586104 PMC3219307

[8] Kamada Y, Tamura S, Kiso S, Matsumoto H, Saji Y, Yoshida Y, et al Enhanced carbon tetrachloride-induced liver fibrosis in mice lacking adiponectin. Gastroenterology. 2003; 125: 1796–1807. 14724832 10.1053/j.gastro.2003.08.029

[9] Ohashi K, Iwatani H, Kihara S, Nakagawa Y, Komura N, Fujita K, et al Exacerbation of albuminuria and renal fibrosis in subtotal renal ablation model of adiponectin-knockout mice. Arterioscler Thromb Vasc Biol. 2007; 27: 1910–1917. 17626903 10.1161/ATVBAHA.107.147645

[10] Fujita K, Maeda N, Sonoda M, Ohashi K, Hibuse T, Nishizawa H, et al Adiponectin protects against angiotensin II-induced cardiac fibrosis through activation of PPAR-alpha. Arteriosclerosis Thrombosis And Vascular Biology. 2008; 28: 863–870. 10.1161/ATVBAHA.107.156687 18309113

[11] Ashcroft T, Simpson JM, Timbrell V. Simple method of estimating severity of pulmonary fibrosis on a numerical scale. J Clin Pathol. 1988; 41: 467–470. 3366935 10.1136/jcp.41.4.467 PMC1141479

[12] Bartram U, Speer CP. The role of transforming growth factor beta in lung development and disease. Chest. 2004; 125: 754–765. 14769761 10.1378/chest.125.2.754

[13] Meyer-Ter-Vehn T, Gebhardt S, Sebald W, Buttmann M, Grehn F, Schlunck G, et al p38 inhibitors prevent TGF-beta-induced myofibroblast transdifferentiation in human tenon fibroblasts. Invest Ophthalmol Vis Sci. 2006; 47: 1500–1509. 16565385 10.1167/iovs.05-0361

[14] Giannandrea M, Parks WC. Diverse functions of matrix metalloproteinases during fibrosis. Dis Model Mech. 2014; 7: 193–203. 10.1242/dmm.012062 24713275 PMC3917240

[15] Atkinson JJ, Senior RM. Matrix metalloproteinase-9 in lung remodeling. Am J Respir Cell Mol Biol. 2003; 28: 12–24. 12495928 10.1165/rcmb.2002-0166TR

[16] Ruiz V, Ordonez RM, Berumen J, Ramirez R, Uhal B, Becerril C, et al Unbalanced collagenases/TIMP-1 expression and epithelial apoptosis in experimental lung fibrosis. Am J Physiol Lung Cell Mol Physiol. 2003; 285: L1026–1036. 12882763 10.1152/ajplung.00183.2003

[17] Keeley EC, Mehrad B, Strieter RM. The role of fibrocytes in fibrotic diseases of the lungs and heart. Fibrogenesis Tissue Repair. 2011; 4: 2 10.1186/1755-1536-4-2 21219601 PMC3027110

[18] Lai CK, Wallace WD, Fishbein MC. Histopathology of pulmonary fibrotic disorders. Seminars In Respiratory And Critical Care Medicine. 2006; 27: 613–622. 17195138 10.1055/s-2006-957333

[19] Krenning G, Moonen JRAJ, Harmsen MC. Pleiotropism of Adiponectin Inflammation, Neovascularization, and Fibrosis. Circulation Research. 2009; 104: 1029–1031. 10.1161/CIRCRESAHA.109.198044 19423859

[20] Luo KX. Ski and SnoN: negative regulators of TGF-beta signaling. Current Opinion In Genetics & Development. 2004; 14: 65–70. 15108807 10.1016/j.gde.2003.11.003

[21] Scotton CJ, Chambers RC. Molecular targets in pulmonary fibrosis—The myofibroblast in focus. Chest. 2007; 132: 1311–1321. 17934117 10.1378/chest.06-2568

[22] Bronnum H, Eskildsen T, Andersen DC, Schneider M, Sheikh SP. IL-1beta suppresses TGF-beta-mediated myofibroblast differentiation in cardiac fibroblasts. Growth Factors. 2013; 31: 81–89. 10.3109/08977194.2013.787994 23734837

[23] Wang Q, Usinger W, Nichols B, Gray J, Xu L, Seeley TW, et al Cooperative interaction of CTGF and TGF-beta in animal models of fibrotic disease. Fibrogenesis Tissue Repair. 2011; 4: 4 10.1186/1755-1536-4-4 21284856 PMC3042008

[24] Brigstock DR. Strategies for blocking the fibrogenic actions of connective tissue growth factor (CCN2): From pharmacological inhibition in vitro to targeted siRNA therapy in vivo. J Cell Commun Signal. 2009; 3: 5–18. 10.1007/s12079-009-0043-9 19294531 PMC2686750

[25] George J, Tsutsumi M. siRNA-mediated knockdown of connective tissue growth factor prevents N-nitrosodimethylamine-induced hepatic fibrosis in rats. Gene Ther. 2007; 14: 790–803. 17344905 10.1038/sj.gt.3302929

[26] Yokoi H, Mukoyama M, Nagae T, Mori K, Suganami T, Sawai K, et al Reduction in connective tissue growth factor by antisense treatment ameliorates renal tubulointerstitial fibrosis. J Am Soc Nephrol. 2004; 15: 1430–1440. 15153554 10.1097/01.asn.0000130565.69170.85

[27] Phan SH. The myofibroblast in pulmonary fibrosis. Chest. 2002; 122: 286S–289S. 12475801 10.1378/chest.122.6_suppl.286s

[28] Pardo A, Selman M. Matrix metalloproteases in aberrant fibrotic tissue remodeling. Proc Am Thorac Soc. 2006; 3: 383–388. 16738205 10.1513/pats.200601-012TK

[29] Lim DH, Cho JY, Miller M, McElwain K, McElwain S, Broide DH. Reduced peribronchial fibrosis in allergen-challenged MMP-9-deficient mice. Am J Physiol Lung Cell Mol Physiol. 2006; 291: L265–271. 16825657 10.1152/ajplung.00305.2005

